# Quantification of the effect of mammographic screening on fatal breast cancers: The Florence Programme 1990–96

**DOI:** 10.1038/sj.bjc.6600301

**Published:** 2002-07-15

**Authors:** E Paci, S W Duffy, D Giorgi, M Zappa, E Crocetti, V Vezzosi, S Bianchi, M Rosselli del Turco

**Affiliations:** Unit of Epidemiology, CSPO, Florence, Italy; Department of Mathematics, Statistics and Epidemiology, ICRF, London, UK; Unit of Epidemiology, ASL Lucca, Lucca, Italy; Pathology Department., University of Florence, Florence, Italy; Breast Unit, CSPO, Florence, Italy

**Keywords:** mammography screening, breast cancer, screening programmes

## Abstract

Breast cancer cases diagnosed in women aged 50–69 since 1990 to 1996 in the City of Florence were partitioned into those who had been invited to screening prior to diagnosis and those who had not. All cases were followed up for vital status until 31 December 1999. The cumulative number of breast cancer deaths among the cases were divided by screening and invitation status, to give the rates of cancers proving fatal within a period of 8 years of observation (incidence-based mortality). We used the incidence-based mortality rates for two periods (1985–86, 1990–96), pre and during screening. The incidence-based mortality ratio comparing 1990–96 and 1985–86 was 0.50 (95% CI : 0.38–0.66), a significant 50% reduction. For noninvited women, compared to 1985-86, there was a 41% significant mortality reduction (RR=0.59, 95% CI : 0.42–0.82). The comparable reduction in those invited was a significant 55% (RR=0.45, 95% CI : 0.32–0.61). The incidence ratio of rates of cancers stage II or worse was close to one when the noninvited in 1990–96 were compared with 1985–86 (RR=0.97, 95% CI : 0.78–1.21). Excluding prevalent cases, the rate of stage II+ breast cancer cases was 42% lower in Screened women compared with the noninvited (RR=0.58, 95% CI : 0.45–0.74). This study confirmed that new treatments and the first rounds of the screening programme contributed to reducing mortality from breast cancer.

*British Journal of Cancer* (2002) **87**, 65–69. doi:10.1038/sj.bjc.6600301 www.bjcancer.com

© 2002 Cancer Research UK

## 

Following the demonstration by randomised clinical trials that mammographic screening reduces mortality for breast cancer, population-based breast cancer screening programmes have been initiated in many countries and the challenge today is to evaluate the effectiveness of these programmes in the routine health care environment. To accomplish this task is difficult, due partly to the many factors influencing the incidence, staging and mortality for breast cancer and partly to the complexity of data collection. Many attempts have been made to analyse breast cancer mortality rates in relation to the introduction of screening programmes. Simple comparisons of mortality rates before and after the introduction of screening is biased by confounding with other changes over time (for example in therapy), and by the inclusion of deaths from tumours diagnosed before the introduction of screening. Blanks *et al* (2000) solved the first of these problems by comparing changes in cohorts likely to have been invited to screening with those in cohorts unlikely to be invited. They noted, however, that the second problem remains, that including deaths from cancers diagnosed before screening was available.

Tabar *et al* (2001) considered deaths in each period only from tumours diagnosed in that period and compared changes over time in age groups exposed to screening invitation with those in age groups which were never invited to screening, thus addressing both problems. In addition, they identified the women who were actually screened and estimated the effects of actually receiving screening as well as of invitation to screening, adjusting for selection bias and lead time. The analysis presented in this paper is based on the data of the Tuscany Tumour Registry which has been operating since 1985 and includes the City of Florence where population-based breast cancer screening programmes started in 1990.

Using standard methods a mortality reduction of 19% for invited women by the screening programme was estimated (Paci *et al*, 2002).

## MATERIALS AND METHODS

Breast cancer cases diagnosed in women resident in the City of Florence were registered by the Tuscany Tumour Registry according to the IARC rules for cancer registration (Berrino *et al*, 1995). Cases ascertained only from death certificates and multiple primaries were excluded. The Florence City screening programme and main performance indicators have been described in detail (Giorgi *et al*, 1994). The target population was the resident female population aged 50–69 years, who were invited over the period from 1990–96 to have 2-view, high quality mammography every 2 years.

All breast cancer cases were linked to the screening database and partitioned by ‘detection’ category as follows:

1. Cases diagnosed in the first round of screening or in a woman's first test at a subsequent round, *viz*. prevalent screen detected cases.
2. Cases detected at repeat screening tests or detected clinically after a negative screening test but before the subsequent invitation or the end of the study period.
3. Cases in non responders.
4. Cases diagnosed in women eligible but not yet invited to screening (since it took several years to achieve full coverage of the population with invitation to screening) or of uncertain diagnostic modality.

In the comparison of mortality of invited and non-invited women, the first three categories were combined to form the invited group and compared with the noninvited. In the comparison of screened with unscreened women, the first two categories only were combined to give the screened group and the second two to give the unscreened. All breast cancer incident cases were followed up for vital status until 31 December 1999 and the underlying cause of death collected. The 50–69 years old female population by calendar year was identified from the records of the municipality of Florence. All resident women in the age range received an invitation in the enrolment period or at the first subsequent round, if eligible. For each woman from January 1990, the starting date of the programme, we calculated the person-years lived before the date of first invitation (noninvited). After the invitation, the women-years at risk of breast cancer (invited) were calculated to the end of the study period (31 December 1996), diagnosis of breast cancer, exit from the target population on reaching the age limit (70th birthday), death or migration. Invited women were further divided into respondents (screened) and non respondents to the invitation. The statistical analysis was performed using Stata (Stata Statistical Software, 2001).

The tumour characteristics were reviewed and size classified as T1a, T1b,T1c or T2+, nodal status as negative or positive and stage according with the UICC-pTNM. Stage II+ tumours were considered as advanced cases. In addition we examined the 5-year cause specific survival by stage. Missing data refer to the non-operated and the unknown. Full data on tumour characteristics were not generally available before 1990. The 1985–86 data had been reviewed for purposes of a previous study and incidence and case- fatality rates specific to the 1990–96 target population were therefore compared to the 1985–86 data (Paci *et al*, 1994). To estimate the impact on mortality we used a method like that of Tabar *et al* (2001) and to the estimate of the Incidence-based mortality (IBM), developed by Chu *et al* (1994). All breast cancer deaths among the cases diagnosed after the first invitation (invited women) and until 31 December 1999, were included in the calculation of the IBM rate, estimated as the rate of fatal incident cases.

**Figure 1:**
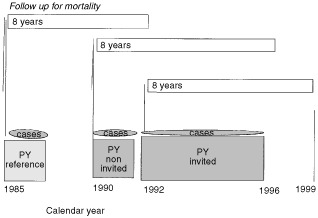
Incidence-based mortality by calendar period and screening status.

Given that enrolment lasted from September 1990 to early 1993, an average duration of observation of 8 years (from 1992–1999) was assumed for invited women. The occurrence of breast cancer deaths within breast cancer cases in 8 years of follow-up was considered for comparison. Deaths from 1990–1997 of noninvited breast cancer cases and from 1985–1992 in cases diagnosed in the period 1985–86, were included in the calculation of the IBM rate. The same 8-year follow-up time from the year of the start of the observation period for all groups was intended to eliminate the lead time bias due to the earlier diagnosis in screened women: breast cancer death rates would have been similar for the three groups in the absence of treatment or screening benefit. (Figure 1

).

The IBM rate is not equivalent to the usual mortality rate because the population at diagnosis rather than at death forms the denominator; in addition, it relates to the age at diagnosis rather than age at death; it differs from the case-fatality rate in that the denominator is population-based and not breast cancer cases. Although not identical to IBM estimates used by Chu *et al* (1994) and applied to interpret trends in prostate cancer in the US (Feuer *et al*, 1999), we refer incidence based mortality (IBM) to our rates below, since IBM seems to be the most accurate description.

The advantages of using the incidence of tumours proving fatal are:

- That this is not affected by length bias or overdiagnosis, since excessive diagnosis of indolent tumours does not affect the number of aggressive tumours; and
- With only two periods, pre-screening and during screening, it is not affected by lead time bias.

## RESULTS

In the period 1990–96, 1122 invasive breast cancers and 84 carcinoma *in situ* cases were diagnosed in women aged 50–69. In the same period of time, 35 568 women received their first screening test and 35 954 a repeat screening test. In 1990–96 there was a total of 419 632 person-years corresponding to approximately 60 000 women each year, invited women had 254 890 and screened women 155 645 person years after their prevalence test to the end of follow-up. The corresponding noninvited person-years were estimated as 164 742. There were 99 425 person-years of observation in women who were invited but did not attend. In the reference period 1985–86, 282 invasive breast cancer were clinically diagnosed in 123 573 women-years.

Table 1

**Table 1:**
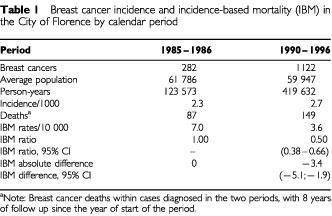
Breast cancer incidence and incidence-based mortality (IBM) in the City of Florence by calendar period

shows the incidence of breast cancer in women aged 50–69 by period. Between the two periods, the breast cancer incidence rate increased from 2.3 to 2.7 per 1000 women aged 50–69. This reflects the peak in incidence in the early years of screening due to the prevalence round. The incidence rates were 3.6 and 2.1 per 1000 in screened and unscreened and 2.9 and 2.3 per 1000 person years, respectively in invited and noninvited women in 1990–96.

The IBM ratio comparing 1990–96 and 1985–86 for fatal breast cancer cases within 8 years since the start of the period was 0.50 (95% CI : 0.38–0.66), a significant 50% reduction corresponding to an absolute difference of −3.4 deaths per 10 000 women years. In Table 2

**Table 2:**
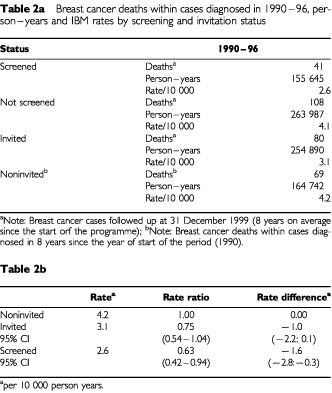
aBreast cancer deaths within cases diagnosed in 1990–96, person–years and IBM rates by screening and invitation status

, deaths from cancers diagnosed in 1990–96, person-years and IBM rates are shown classified by screening and invitation status. The IBM rates were 25% less (RR=0.75, 95% CI : 0.54–1.04) for invited compared to noninvited women: and the reduction was statistically significant for screened women (RR=0.63, 95% CI : 0.42–0.94). To estimate the trend in mortality occurring independently of screening, we calculated the reduction in the IBM in noninvited women compared to 1985–86, there was a 41% significant reduction (RR=0.59, 95% CI : 0.42–0.82). The comparable reduction in those invited was 55% (RR=0.45, 95% CI : 0.32–0.61).

The reduction in breast cancer mortality is related to more favourable stage distribution and to improved breast cancer specific survival rates overall and by stage, presumably due to better treatment and breast cancer care. Incidence rates of advanced tumours are considered good indicators of the mortality reduction (Tabar *et al*, 2001). In order to account for the incidence inflation of cases due to the prevalence screening, rates are presented separately for cases screen-detected at the prevalence test (the exclusion of prevalent cases is marked with * in screened*, invited* and total* groups). Cases diagnosed at the prevalent screen are most prone to lead time and over-diagnosis biases and not occur as incident cancer cases within the study period. The 5-years cause-specific survival rates improved from 75% for the 1985–86 breast cancer cases to 90% in invited (including screen detected cases) and 84% in noninvited women diagnosed in the period 1990–1996.

Forty-two per cent of stage II+ breast cancer cases were detected earlier by screening in screened* women compared with the noninvited (RR=0.58, 95% CI : 0.45–0.74); 28% fewer stage II+ tumours were observed in the invited* compared with the noninvited (RR=0.72, 95% CI : 0.59–0.87) (Table 3

**Table 3:**
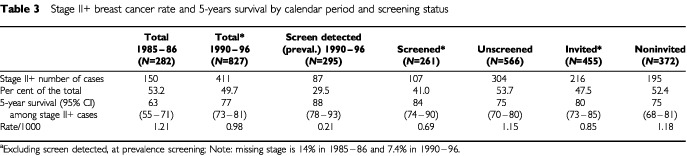
Stage II+ breast cancer rate and 5-years survival by calendar period and screening status

). The 5-year survival rate was 88% for stage II+ breast cancers detected at the prevalence screening, suggesting a within-stage lead time. The corresponding survival rates for stage I cancers were also increased (data non shown). In the total* group, i.e. excluding cases screen detected at prevalence, the stage II+ disease rate was reduced by 19% (RR=0.81, 95% CI : 0.67–0.98), but the ratio was near to one when the noninvited women only were compared with the 1985-86 rate (RR=0.97, 95% CI : 0.78–1.21). The corresponding 5-year survival rates for stage II+ increased significantly from 63% in 1985–86 to 75% in 1990–96 in noninvited women.

Table 4

**Table 4:**
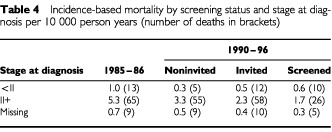
Incidence-based mortality by screening status and stage at diagnosis per 10 000 person years (number of deaths in brackets)

shows the comparison of the IBM rates for 1985–86 with the noninvited and invited women by stage (<II;II+; missing). In breast cancer cases stage II+ at diagnosis, the IBM rate difference was −1.9 per 10 000 person-years (RR=0.63, 95% CI : 0.43–0.92) between the rate observed in 1985-86 and the rate of noninvited women; a reduction of −1.1 breast cancer deaths per 10 000 person-years was estimated between invited and noninvited women (RR=0.68, 95% CI : 0.46–1.00). For breast cancer cases staged as less than II at diagnosis, the IBM rate showed a reduction of −0.75 deaths per 10 000 between the 1985–86 cases and the noninvited. The IBM rate from stage less than II cancers in the noninvited women was higher than that in the invited, although not significantly so (RR 1.55, 95% CI : 0.51–5.62).

## DISCUSSION

The main aim of this paper was to develop a method for evaluating population-based breast cancer screening programmes using data on incidence, survival and staging, and by linking cancer registry data with a minimum set of data from the screening services. The use of IBM measures should prove useful in future evaluation with longer follow up. A summary of the changes in various IBM and stage II+ rates between 1985–86 and 1990–96 are given in Table 5

**Table 5:**
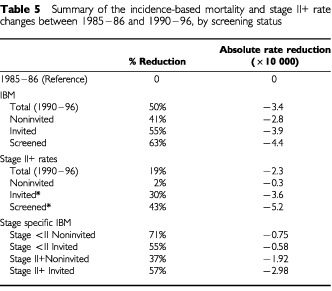
Summary of the incidence-based mortality and stage II+ rate changes between 1985–86 and 1990–96, by screening status

. The incidence of fatal tumours fell significantly by 41% between the 1985–86 reference and the noninvited women, who were diagnosed after 1990. The IBM rate difference between 1985–86 and the noninvited was −1.92 per 10 000 women with a stable rate of stage II+ tumours. The increase in survival rates, comparing the late 1980's with early 1990's has been documented by several cancer registries (Rosso *et al*, 2001). The 5-years breast cancer survival increased by 9% when 1985–86 and noninvited breast cancer cases are compared. Although a shift towards earlier diagnosis cannot be excluded, this result is a major confirmation of the impact of changes in breast cancer therapy and care on breast cancer mortality. Assuming, as estimated by Chu *et al* (1994), an 8 years lag required for the IBM rates to explain the overall breast cancer mortality data, the observed IBM rate reduction is probably in good agreement with the reduction of breast cancer mortality observed in Florence City in the early 1990s (Barchielli and Paci, 2001).

The reduction from the reference period of −3.9 deaths per 10 000 in the invited (and the −4.4 per 10 000 less deaths amongst the screened) when compared with the −2.8 death reduction in the noninvited, indicates that about one-third of the reduction in women exposed to screening is due to the screening rounds and the other two-thirds due to other causes, including therapeutic innovations. The stage II+ rate reduction for the screened* (−5.2 advanced cases per 10 000) suggests that the effect might increase as the screening process continues and with longer follow up. These results differ from those of Tabar *et al* (2001), though the relative reduction of mortality in the screened group is similar. That study implied that the majority of the mortality reduction was attributable to screening. In our study the period of observation is short and the start of screening followed major changes in therapy in the eighties.

The proportion of all breast cancer cases which were detected at screening in the period 1990–96 was in total about 40% of all cases and the person-years of screened women in this period were 155 645 (37% of the total). In order to estimate the number of deaths saved, we applied the IBM rates in the reference period (1985/86), for noninvited and invited to the screened women years. One hundred and eight breast cancer deaths within incident cases would have been expected applying the IBM rate of the reference period, and 65 by applying the noninvited IBM rate. Considering the expected number of incident cases (incidence rate of 2.3 per 1000), 358 breast cancer incident cases were expected in the absence of screening. The reduction in the number of deaths is quite comparable to the reduction in the case-fatality rates, though calculated by a different method. The estimate of the expected number of deaths using the invited women IBM rate was 49, indicating that the number of deaths saved compared with the expected applying the noninvited IBM rate, of 16 (intention-to-treat analysis); applying the IBM rate of screened women gives the estimated number of lives saved as 24. Among the 35 568 women in the first screening 0.45 deaths were saved per 1000 screened women if the rate of the invited is applied or 0.67 if based on the screened women. This result is in agreement with the risk reduction expected from screening 8 years from the beginning. Further follow-up will allow more reliable evaluation of the impact of the screening programme.

With regard to the evaluation of the IBM rates by stage, the comparison of the 1985–86 IBM rates with the noninvited and invited in 1990–96 showed a reduction of fatal cancer deaths in cases diagnosed as advanced. This finding might be attributable to the improvement of the case-fatality rate because of better treatment or to a reduction of the rate of stage II+ cancers. The stage <II IBM rate decreased for noninvited and the invited in comparison with the reference period, but a non-significant increase of the early stage IBM rate was observed between invited and noninvited. A possible stage shift bias between advanced and less advanced carcinomas might determine an increase of the IBM rate of less advanced cases. The small increase so far observed excludes an important misclassification bias of advanced tumours related to the detection at screening.

Recently breast cancer mortality rates have been used to estimate the possible mortality reduction related to screening in England and Wales. The result was a 6.4% reduction in overall breast cancer mortality which was attributed to screening, approximately one-third of a 20% reduction (Blanks *et al*, 2000). This estimate, however, was based on modelling breast cancer deaths regardless of period of diagnosis. Thus deaths from tumours diagnosed before screening was introduced were included in the cohorts nominally exposed to screening. It would be interesting to see the results for England and Wales with respect to IBM. Our data shows that treatment and screening have reduced breast cancer mortality in Florence, but the full impact of the screening programme needs a longer follow up and improved programme performance (compliance rates, interscreening interval). However, we consider that our incidence-based method for the monitoring of the efficacy of breast cancer screening programmes is useful and it will be complemented by an evaluation based on the individual screening history. The collection of details of the screening history for each individual can be difficult. Irregular participants (and therefore irregular non-participants) can represent a large proportion of the population. For this reason, an approach based on sampling, such as a case-control study, is a possible tool to corroborate the results presented in this paper and is currently under investigation.
